# Investigating the Proton and Ion Transfer Properties of Supported Ionic Liquid Membranes Prepared for Bioelectrochemical Applications Using Hydrophobic Imidazolium-Type Ionic Liquids

**DOI:** 10.3390/membranes11050359

**Published:** 2021-05-14

**Authors:** László Koók, Piroska Lajtai-Szabó, Péter Bakonyi, Katalin Bélafi-Bakó, Nándor Nemestóthy

**Affiliations:** Research Group on Bioengineering, Membrane Technology and Energetics, University of Pannonia, Egyetem ut 10, 8200 Veszprém, Hungary; kook@almos.uni-pannon.hu (L.K.); tpiroska94@gmail.com (P.L.-S.); bako@almos.uni-pannon.hu (K.B.-B.); nemesn@almos.uni-pannon.hu (N.N.)

**Keywords:** ionic liquid, SILM, membrane, proton transfer, ion transport, DC conductivity

## Abstract

Hydrophobic ionic liquids (IL) may offer a special electrolyte in the form of supported ionic liquid membranes (SILM) for microbial fuel cells (MFC) due to their advantageous mass transfer characteristics. In this work, the proton and ion transfer properties of SILMs made with IL containing imidazolium cation and [PF_6_]^−^ and [NTf_2_]^−^ anions were studied and compared to Nafion. It resulted that both ILs show better proton mass transfer and diffusion coefficient than Nafion. The data implied the presence of water microclusters permeating through [hmim][PF_6_]-SILM to assist the proton transfer. This mechanism could not be assumed in the case of [NTf_2_]^−^ containing IL. Ion transport numbers of K^+^, Na^+^, and H^+^ showed that the IL with [PF_6_]^−^ anion could be beneficial in terms of reducing ion transfer losses in MFCs. Moreover, the conductivity of [bmim][PF_6_]-SILM at low electrolyte concentration (such as in MFCs) was comparable to Nafion.

## 1. Introduction

MFCs belong to the family of bioelectrochemical technologies, where the bioelectrocatalytic activity of exoelectrogenic bacteria (EAB), which are known for their ability to transfer electrons extracellularly to the surface of an electrode, is utilized [1,2]. MFCs usually consist of an ion permeable membrane separating the electrode chambers. Generally, the membrane is proton selective (e.g., Nafion) and its main purpose is to ensure sufficient proton transport from the anode to the cathode for the reduction of an electron acceptor (most often O_2_) in line with Equation (1).

O_2_ + 4e^−^ + 4H^+^ → 2H_2_O (1)

However, the low proton concentration in MFCs ([H^+^] < 10^−6^ M) inherently cause deviations from Equation (1) at pH values close to neutral [3] and instead, the cathodic reaction formulated in Equation (2) takes place, foreshadowing the increase of catholyte pH (to 10–13) due to OH^−^ formation.

O_2_ + 4e^−^ + 2H_2_O → 4OH^−^ (2)

In addition, a general anolyte with the inocula, the buffer, and the feedstock, e.g., wastewater, contains an amount of other cations (mainly Na^+^, K^+^, Mg^2+^, Ca^2+^) 4–5 orders of magnitude higher than protons. Thus, proton transfer across the membrane can be surpassed and as a result, H^+^ accumulates in the vicinity of the anode, the pH locally drops, and the inhibition of EAB may occur along with the limitation of electricity generation. Therefore, the development of membranes with improved charge transfer characteristics for bioelectrochemical applications such as MFCs is still a challenge. Ionic liquids (ILs) are often referred to as the green solvents and electrolytes of the future due to their excellent properties, such as negligible vapor pressure, low volatility, non-flammability, excellent thermal stability, flexible solvation features, and tunability by varying the cation/anion pairs [4,5,6,7,8]. Among others, they have been successfully used as non-conventional solvents for synthetic, hydrolytic, polymerization, (bio)catalytic, etc. processes [9,10,11]. Their use in electrochemistry focuses mostly on the heterogeneous electron-transfer processes, thanks to their good conductivity, wide viscosity range, and great electrochemical stability [12,13]. Moreover, ILs are used in separation technology in different forms, including membranes [14,15,16]. In fact, membranes containing imidazolium-type ILs could be applied effectively in MFCs by fabricating supported or polymer inclusion membranes [17,18,19]. It resulted that, compared to Nafion, SILMs with hydrophobic hexafluorophosphate and bis(trifluoromethylsulfonyl)imide ([PF_6_]^−^ and [NTf_2_]^−^) anions and 1-butyl- or 1-hexyl-3-methylimidazolium ([bmim]^+^ and [hmim]^+^) cations have beneficial features, including lower acetate mass transfer and diffusion coefficients, as well as lower oxygen permeation (in case of [NTf_2_]^−^) [20]. It was demonstrated that MFC with SILMs based on [bmim][PF_6_] were able to outperform the Nafion-equipped ones, due to the more advantageous mass transfer-related properties (e.g., lower diffusion resistance and cathodic overpotential) [21]. Another aspect that highlights the inherent potential of ILs in this field is their antimicrobial effect [22,23]. This feature could play a key role in, e.g., the mitigation of biofouling, and thus, supporting more stable MFC operation and minimizing cross-membrane mass transfer-related losses. Nevertheless, this aspect should consider the IL dissolution from the membrane pores, which underlines the importance of sufficient SILM stability.

In this work, SILMs based on imidazolium-type ILs bearing [PF_6_]^−^ and [NTf_2_]^−^ anions were investigated in terms of proton transfer characteristics, transport numbers, and ionic conductivity. The results were compared to Nafion as a reference material. To the best of our knowledge, such an approach to describe the possible proton and ion transfer behavior of SILMs made with these ILs is presented for the first time.

## 2. Materials and Methods

### 2.1. Supported Ionic Liquid Membrane Preparation

The SILMs were prepared as described in our previous works [19,20]. Briefly, the ILs were kept under vacuum for at least 2 days prior to use in order to get rid of water contaminants. The supporting hydrophobic PVDF with 0.22 μm pore size and 75% porosity (Durapore) was pretreated under vacuum for 1 h, then, still under vacuum, the IL (ca. 2 mL) was injected to the surface of it. After at least 2 h of vacuuming, the surface of the obtained membranes was gently cleaned using parchment paper, and then, the membranes were weighted. In this research, [bmim][PF_6_], [hmim][PF_6_], and [bmim][NTf_2_] ILs were immobilized in the pores of the PVDF membrane. The main physical properties of the applied ILs can be seen in Table 1. The weight loss of ILs after vacuuming was determined, and it was systematically higher for [PF_6_]^−^ containing ILs (~3-times greater loss) compared to the [NTf_2_]^−^-based IL, which is in agreement with the differences in hygroscopic character of the ILs. The membranes were weighted before and after IL immobilization (and excess IL removal), and the amount of IL immobilized was found to be independent of the IL type; ~300 mg of all ILs could be introduced into the supporting membrane, resulting in 11–11.5 mg cm^−2^ IL content relative to the membrane surface area. This value fits well with previously published literature data [18,24].

### 2.2. Proton Transfer Characteristics

Prior to the experiments, the Nafion115 proton exchange membrane (PEM) was pre-conditioned as described elsewhere [27]. A two-chambered glass reactor was deployed in the mass transfer measurements. The chambers were separated by the actual membrane (kept in deionized water for 1 h prior to the measurements), with a 9.62 cm^2^ surface area. The membranes from both sides were in contact with 55–55 cm^3^ liquid. Initially, one half-cell was filled with deionized water (catholyte, pH = 7), while the other (anolyte) was filled with deionized water and then its pH was set to 8.5 using NaOH [28]. The pH of the anolyte was continuously monitored by using a pH meter until one unit of pH change was recorded. Then, the proton mass transfer coefficient (k_H+_) was calculated according to Equation (3) [28,29],

(3) $$k_{H+}=-\frac{V}{2\;A\;t}\ln\left(\frac{c_{1,0}+c_{2,0}-2c_{2}}{c_{1,0}}\right)$$

where V is the volume, A_M_ is the membrane’s surface area, c_1,0_, c_2,0_, and c_2_ are the proton concentrations initially in the catholyte and anolyte, and in the anolyte at time t, respectively. The proton diffusion coefficient (D_H+_) can be calculated by taking into account the thickness of the actual membrane (d) (Equation (4)).

(4) $$D_{H+}=k_{H+}\;d$$

Considering D_H+_, and the electric charge of a proton (q_H+_ = 1.02 × 10^−19^ C), the proton mobility (μ_H+_) was derived from the Einstein-equation (Equation (5)) [30].

(5) $$\mu_{H+}=\frac{D_{H+}{\;q}_{H+}}{k_{B\;}T}$$

### 2.3. Transport Numbers and Conductivity

To determine the transport numbers of K^+^, Na^+^, and H^+^, a H-cell was assembled with 250–250 mL effective chamber volumes. The given membrane was placed between the compartments. A concentration gradient was applied between the two sides of the membrane (c_1_ = 0.05 M and c_2_ = 0.01 M) by using KCl, NaCl, or HCl solutions. An Ag/AgCl (3 M KCl) reference electrode was placed in each chamber close to the membrane surface and the potential difference (φ) between these two electrodes was measured by a digital multimeter. Once φ reached a plateau (indicating the end-point of the test), the transport number for the i ion (t_i_) was calculated according to Equation (6) [31],

(6) $$t_{i}=\frac{\left\{\frac{∆\phi \;F}{R\;T\;\ln\left(\frac{c_{1}}{c_{2}}\right)}+1\right\}}{2}$$

where R is the universal gas constant, F is the Faraday’s constant, and T is the temperature.

**Figure 1:**
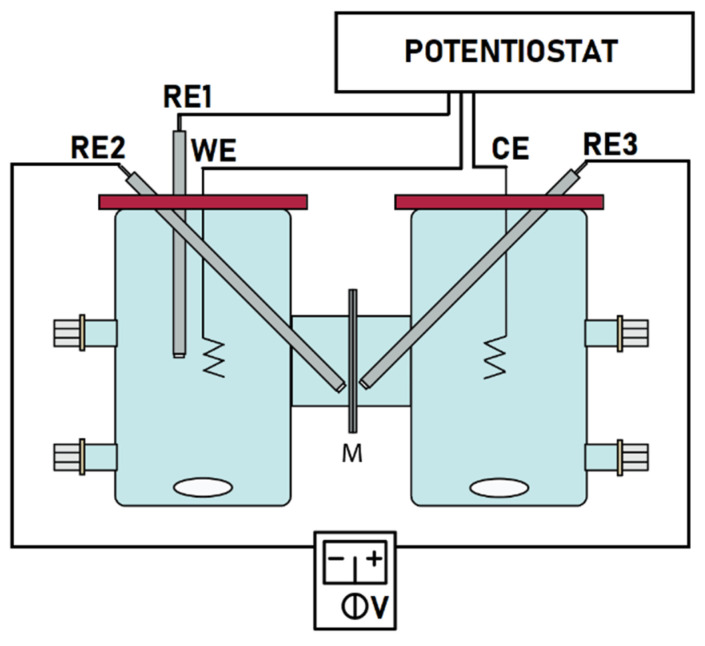
Experimental setup for chronopotentiostatic DC conductivity measurements.

The DC conductivity measurements required a slightly more sophisticated setup (Figure 1), where, in addition to the two Ag/AgCl reference electrodes at the two sides of the membrane (RE2 and RE3), both chambers were installed with a 2 cm long spiral Pt wire electrode (0.5 cm diameter and 2 mm wire thickness), which played the roles of the working (WE) and counter electrodes (CE), respectively. Another Ag/AgCl was placed next to the working electrode (RE1) in order to accomplish chronopotentiostatic control of the current flowing through the membrane, employing a PalmSens3 potentiostat/galvanostat (PalmSens BV, Houten, The Netherlands).

The covered range of current density (relative to the membrane surface area) was 10^−3^–1 mA cm^−2^ in accordance with practical MFC current densities [32]. The φ, similarly to the transport number measurements, was registered by a digital multimeter. The experiments were carried out at various electrolyte (KCl) concentrations from 0.05 M to 0.5 M. The electrolyte solutions were continuously stirred during the tests (100 rpm). To obtain the ionic conductivity, the experiments were performed in the presence and absence of the membrane. The slope of the current- φ linear plot for the membrane-less conditions was derived from the membrane + electrolyte slopes. Conductivity (κ) was then calculated according to Equation (7):

(7) $$\kappa ={\left(\frac{∆\phi {\;A}_{M}}{I\;d}\right)}^{-1}$$

where A_M_ is the membrane surface area, d is the membrane thickness, and I is the current.

## 3. Results and Discussion

### 3.1. Proton Mass Transfer Characteristics

**Figure 2:**
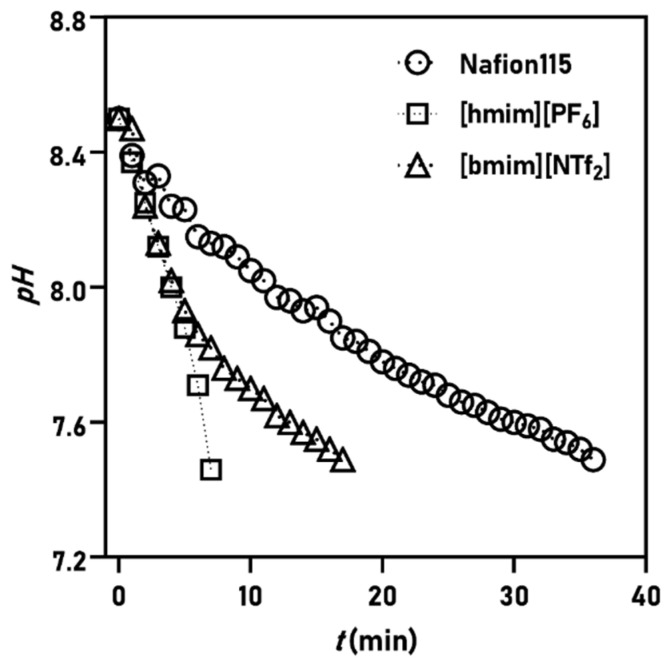
The change in anolyte pH over time using different membranes.

The SILMs with ionic liquids of different properties, e.g., viscosity (Table 1), were prepared, in which the capillary forces arising in the pores influenced SILM stability and thus, the global mass transfer traits during operation. The rate of change in pH was the slowest in the case of Nafion, and the SILMs showed faster pH decrease (Figure 2). The one unit drop of pH could be observed after 7, 17, and 36 min in the cases of [hmim][PF_6_]-SILM, [bmim][NTf_2_]-SILM, and Nafion, respectively. As for Nafion, a smooth linear pH decrease could be observed, while the two SILMs showed a slightly different time-course. By using [bmim][NTf_2_]-SILM, the rate of pH-shift apparently became more and more moderated over time after the first 6 min. Meanwhile, after several minutes of operation, the [hmim][PF_6_]-SILM seemed to ensure enhanced proton transfer kinetics.

**Figure 3:**
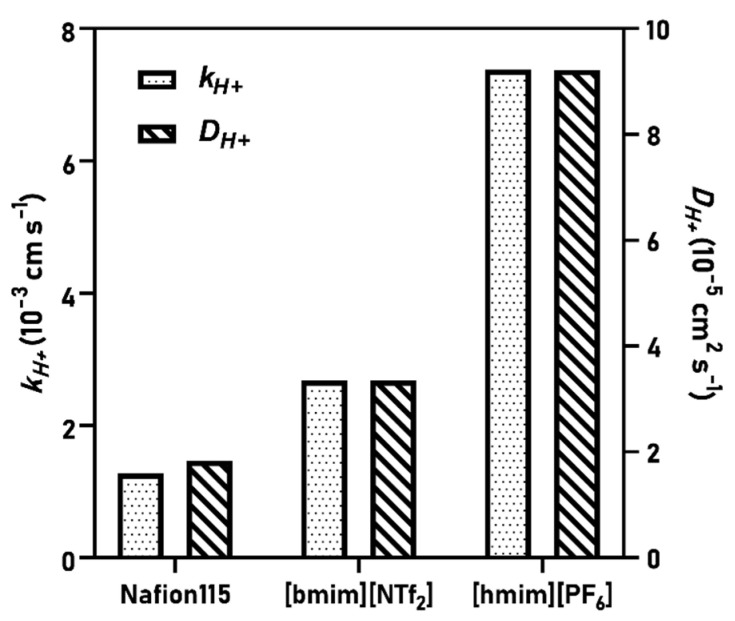
Proton mass transfer and diffusion coefficient values of different membranes.

Based on k_H+_ and D_H+_, the lowest values were indeed found for Nafion, while the k_H+_ and D_H+_ were 2-times and 80% higher for [bmim][NTf_2_]-SILM and [hmim][PF_6_]-SILM, respectively. The results of [hmim][PF_6_]-SILM significantly exceeded the ones obtained with the other two membranes (Figure 3). These outcomes lead to several conclusions. On the one hand, the two SILMs could act as liquid electrolytes and increase the proton transfer between the anolyte and catholyte. This finding can be seen as an advantageous characteristic, in addition to the beneficial acetate and oxygen mass transfer features reported in previous publications [19,20]. On the other hand, by further exploring the particularly high mass transfer features of [hmim][PF_6_]-SILM, the mechanism of protons transfer can be understood. Based on D_H+_ (D_H+_ = 9.2 × 10^−5^ cm^2^ s^−1^), it can be noted that it makes a good match with the one measured in water at the relevant temperature (D_H+_ = 9.3 × 10^−5^ cm^2^ s^−1^) [33,34]. Moreover, in the [hmim][PF_6_]-SILM, μ_H+_ = 3.59 × 10^−7^ m^2^ V^−1^ s^−1^ was obtained, which coincides well with μ_H+_ measured in water (3.62 × 10^−7^ m^2^ V^−1^ s^−1^) [30,35].

This finding indicates that water permeates through the IL and the proton transfer through the SILM is mediated by water diffusion. This is quite concurrent with previous observations in literature, where it was shown that after achieving critical water concentration in the IL, continuous water permeation could occur by microclusters through imidazolium-type ILs with [PF_6_]^−^ anion [36,37,38]. Thus, it can be deduced that protons in this IL are transferred via water microclusters as a result of the (low, but still existing) miscibility of water with the IL [39]. Among non-selective separators, this phenomenon seems quite usual, as can be seen in Table 2 where data from this work and literature sources for different separators demonstrate that proton diffusion coefficients and electric mobilities are in agreement with the values valid in water [28,40].

It was shown previously that the [NTf_2_]^−^ anion has a more hydrophobic character compared to [PF_6_]^−^ [41,42,43,44]. Moreover, usually the hydrophobicity is determined in a greater extent by the properties of anions rather than those of cations (e.g., slight difference in alkyl chain length) [44,45,46,47]. As for the [bmim][NTf_2_], with more significant hydrophobicity, it is assumed that water microcluster formation in the IL phase does not occur. This seems to be in line with the measured k_H+_, D_H+_, and μ_H+_, which are notably lower relative to [hmim][PF_6_]. Furthermore, as D_H+_ is two orders of magnitude higher than the diffusivity of water in [bmim][NTf_2_] [48], the transfer of protons via water microclusters is unlikely. Since [bmim][NTf_2_] is an aprotic IL, the role of the acidic proton of the organic cation’s imidazole ring (position 2) should not be significant (no proton exchange in the aprotic imidazole ring is presumed) [49,50], which was supported by NMR tests of [bmim][NTf_2_]-SILM being contacted with deuterated acetic acid (no observable H^+^–D^+^ exchange at the spectra, data not shown). Based on this, although the question of the exact proton transfer mechanism through [bmim][NTf_2_] still remains open, it can at least be stated that the role of [NTf_2_]^−^ anion could be important in that matter. It was already proposed that the ion transfer in aprotic ILs proceeds via vehicle mechanism, and that the anisotropic cation-anion structure in [bmim][NTf_2_] results in a wider free space for anion motion, which could play a role in proton transfer [51,52,53]. However, many of the conclusions are based solely on computational data, and in order to understand the mechanism, further research is needed. Nevertheless, it was shown that both ILs used in this work as SILMs ensured better proton transfer characteristics than Nafion.

### 3.2. Cation Transport Numbers and Conductivity

As the IL with [PF_6_]^−^ anion demonstrated significantly higher k_H+_ and D_H+_, it is worth investigating how these features support the MFC efficiency in a complex and varying cross-membrane ion transfer environment. As it was underlined, the high concentration of cations induces their transfer through the membrane to accomplish charge-balancing, and protons can be solely transported when it becomes energetically preferred. Accordingly, understanding the ion transfer behavior of the IL together with proton transfer characteristics could be useful to explain the notable efficiency of MFCs operated with SILM [21].

**Figure 4:**
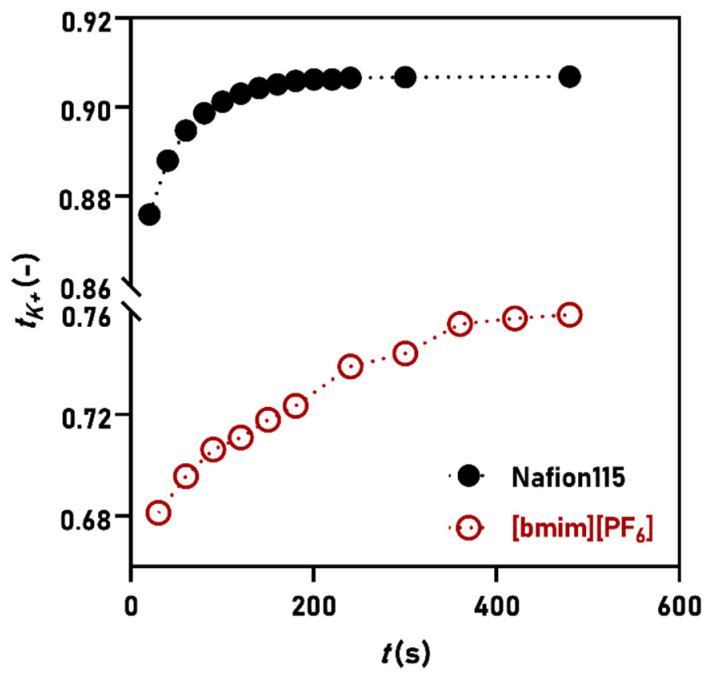
Time-course of K^+^ transport numbers in the case of Nafion and [bmim][PF_6_] membranes.

Therefore, ion transport numbers were sought for Nafion and SILM prepared with [bmim][PF_6_]. The slight reduction in the imidazolium cation’s alkyl chain length compared to [hmim]^+^ was applied to ensure easier SILM preparation (reduced IL viscosity) and further facilitate the diffusion of certain compounds (such as water). The transport numbers were determined for Na^+^, K^+^, and H^+^. In Figure 4, the stabilization of the K^+^ transport number using Nafion and [bmim][PF_6_]-SILM is shown and the difference between t_K+_ values seem significant. A result of t_K+_ ≈ 0.91 was obtained for Nafion, which is in good agreement with its high permselectivity. However, the [bmim][PF_6_]-SILM showed t_K+_ ≈ 0.75, indicating that Cl^−^ ions have notably contributed to the ion transport.

Additionally, this value implies that the diffusion of ions through the SILM is not driven by any ion-selective feature of the [bmim][PF_6_] and rather, it is proportional to the size of the ions in the electrolyte (the ratio of ion radii for K^+^ and Cl^−^ is r_K+_/r_Cl−_ = 0.762). This supports the assumption that protons—and consequently ions—transfer by the assistance of water through the IL with [PF_6_]^−^.

Considering the transport numbers, Nafion reflected higher values in all cases (Table 3). The [bmim][PF_6_]-SILM exhibited a lower transport number for Na^+^ (t_Na+_ = 0.761), whilst for H^+^, it could achieve t_H+_ as high as 0.933 (t_H+_ = 0.978 for Nafion). As already stressed, MFCs equipped with Nafion may suffer from remarkable diffusion losses related to cross-membrane transfer processes [21,54]. This can be attributed to the fact that these cations migrate through the membrane instead of protons in most of the current generation phase. Nafion has great cation permselectivity, which in MFCs may be a drawback, as the rate of the ion transfer limits the performance. The findings presented so far reveal that [bmim][PF_6_]-SILM could be advantageous for MFCs, since the passage of anions such as Cl^−^ is also significant in addition to cation transfer, unlike in the case of Nafion, which may result in an increase in ion transfer rate coupled with a reduction in ion transfer losses. Moreover, the transport numbers revealed that in the sole H^+^-transferring stage of the MFC operation, [bmim][PF_6_]-SILM can satisfy the requirements of a proton exchange membrane (t_H+_ > 0.9).

**Figure 5:**
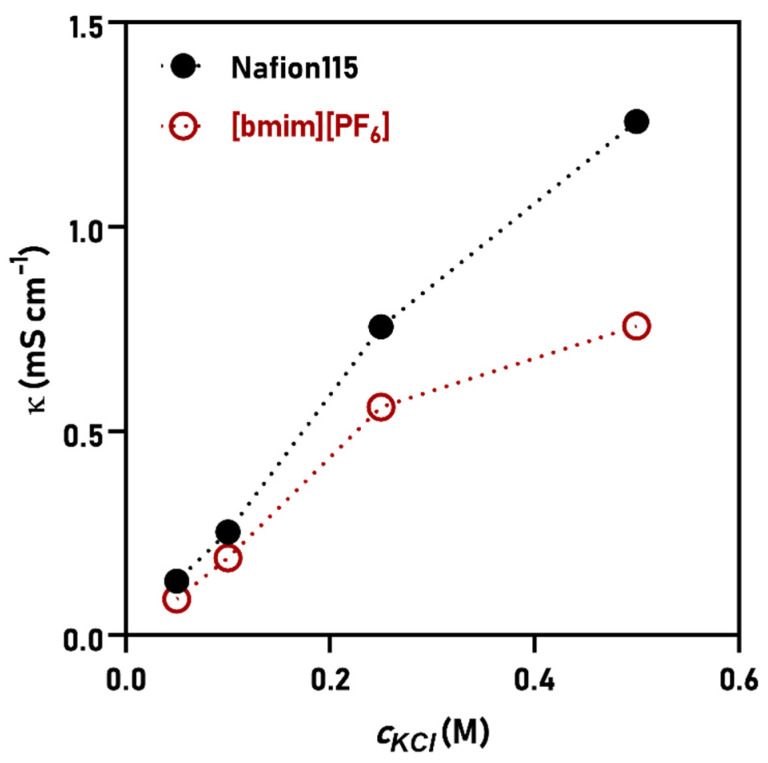
The dependence of conductivity on the electrolyte concentration in the cases of Nafion and [bmim][PF_6_]-SILM.

Another feature that novel membrane materials should possess is sufficient ionic conductivity. MFCs represent a special platform in this context, since the conductivity of electrolytes is low, usually varying between 0.06 and 1 S m^−1^, depending on the source of the electrolyte [3]. This means that membranes ought to work efficiently even at low ion concentration. Most commercial cation exchange membranes are characterized at a standard experimental point in terms of conductivity, using 0.5 M KCl or NaCl. Furthermore, it is known that membrane conductivity can significantly decrease by lowering the concentration, and in MFCs, the membranes are challenged by this issue. By addressing the conductivity of Nafion and [bmim][PF_6_]-SILM at different electrolyte concentrations, we could conclude the superior conductivity of Nafion only at high KCl concentrations (*c_KCl_* > 0.1 M) (Figure 5).

Below that—i.e., by approaching real MFC conditions—the κ of the two membranes tend to approach each other. Consequently, on the grounds of κ, [bmim][PF_6_]-SILM becomes comparable to Nafion, which coincides with the result of our previous research, where impedance spectra unveiled similar impedances at high frequencies for Nafion and [bmim][PF_6_]-SILM during MFC operation [21].

## 4. Conclusions

In this work, SILMs were fabricated using imidazolium-type ILs to investigate their proton and ion transfer traits. Based on the outcomes, the underlying mechanisms of proton transfer could be distinguished for imidazolium-type ILs with [PF_6_]^−^ and [NTf_2_]^−^ anions and both SILMs were competitive with Nafion as shown by k_H+_ and D_H+_. The water permeation through the IL with [PF_6_]^−^ allows enhanced charge transfer kinetics, and in light of the transport numbers determined for K^+^, Na^+^, and H^+^, it seems to counteract the negative effects originating from the non-ideal ion transfer processes taking place in MFCs. The transport numbers obtained for [bmim][PF_6_]-SILM demonstrated that it may serve as an advantageous separator in real MFCs, thanks to its non-selective charge transfer (ion size proportional diffusion) and sufficient conductivity at low electrolyte concentrations.

## Figures and Tables

**Table 1:** Basic data and structure of the applied ILs.

Property	[bmim][PF_6_]	[hmim][PF_6_]	[bmim][NTf_2_]
Molar mass (g mol^−1)^	284.18	312.24	419.36
Density (g cm^−3^)	1.37 *	1.2932 **	1.4366 **
Viscosity (mPa s)	273 *	496.4 **	50.9 **
Structure of cations	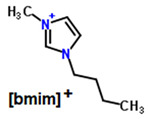 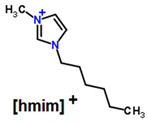		
	9.29		
	3.62		
Structure of anions	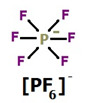 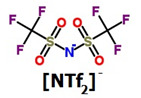		

* Data based on Ref. [25], measured at 25 °C and 0.1 MPa. ** data based on Ref. [26], measured at 25 °C and 0.1 MPa.

**Table 2:** Proton mass transfer coefficient, diffusion coefficient, and electric mobility of various membranes.

Membrane	k_H+_ (10^−3^ cm s^−1^)	D_H+_ (10^−5^ cm s^−1^)	μ_H+_ (10^−7^ m^2^ V^−1^ s^−1^)	Ref.
Nafion115	1.27	1.83	0.713	This work
[bmim][NTf_2_]	2.68	3.35	1.30	
[hmim][PF_6_]	7.38	9.22	3.59	
CMI-7000	2.02	9.29	3.62	[40]
UFM	2.82	9.31	3.63	
SPEEK	4.66	9.32	3.63	
glass fiber	0.94	9.40	3.66	[28]
textile	30.27	9.08	3.54	

**Table 3:** Transport numbers of various ions in the cases of Nafion and [bmim][PF_6_]-SILM.

Membrane	t_K+_	t_Na+_	t_H+_
Nafion115	0.907	0.910	0.978
[bmim][PF_6_]	0.747	0.761	0.933

## Data Availability

Data associated with this research are mentioned in the article.
